# The role of allostatic load in adverse pregnancy outcomes: a multisystem, developmental perspective

**DOI:** 10.3389/fgwh.2025.1725275

**Published:** 2026-01-08

**Authors:** Lauren A. Costello, Sarah M. Banker, Santiago Morales, Maria Barber, Christine Hockett, Lacey McCormack, Virginia A. Rauth, Amy J. Elliott, Lauren C. Shuffrey

**Affiliations:** 1 Department of Child and Adolescent Psychiatry, NYU Grossman School of Medicine, New York, NY, United States; 2 Department of Psychology, Northeastern University, Boston, MA, United States; 3 Department of Psychology, University of Southern California, Los Angeles, CA, United States; 4 Avera Research Institute, Sioux Falls, SD, United States; 5 Department of Pediatrics, University of South Dakota School of Medicine, Sioux Falls, SD, United States; 6 Heilbrunn Department of Population and Family Health, Mailman School of Public Health at Columbia University, New York, NY, United States

**Keywords:** adverse pregnancy outcomes, allostasis, allostatic load, chronic stress, offspring, pregnancy

## Abstract

Allostatic load provides a valuable framework for examining how cumulative stress impacts multiple physiological systems simultaneously, making it a powerful tool for understanding the mechanisms through which stress contributes to adverse pregnancy outcomes. This multisystem perspective is especially important during pregnancy, a period characterized by heightened vulnerability to stress and significant physiological changes that can themselves contribute to allostatic load. Although the impact of allostatic load during pregnancy is well documented, the mechanisms and moderators involved by trimester remain unclear, particularly given wide variation in social, cultural, and structural determinants of maternal health worldwide. In this review, we discuss the progress that has been made over the past two decades in studying prenatal allostatic load and describe the clinical implications of this by highlighting sensitive periods of interest throughout gestation. Despite these advances, key questions remain regarding the intergenerational transmission of risk, the specificity of findings to the pregnancy period, and the role of factors that often accompany elevated allostatic load, such as poor sleep, limited social support, systemic inequities, and comorbid mental or physical health conditions, which may manifest differently across global contexts. Most existing studies have been conducted in high-income settings, yet the burden of adverse pregnancy outcomes is greatest in low- and middle-income countries, where structural, environmental, and social stressors are pervasive. Expanding this framework to include diverse global contexts is essential for understanding how social inequities and chronic stressors shape maternal physiology worldwide. We discuss these issues and offer directions for future research, including the goal of developing a standardized metric for measuring allostatic load – one that we believe will advance the field's understanding of how prenatal allostatic load markers by trimester relate to maternal and infant outcomes.

## 1 What is allostatic load and why is it important?

Stress is conceptualized as a process that involves recognition of and response to threat or danger (1) that can initiate a cascade of physiological changes throughout the body. The unique variations in how the brain interprets environmental stressors and how the body reacts to them ultimately determine whether one is vulnerable to stress or resilient against it (2–4). Biological processes are likely honed by evolution to prioritize survival, favoring stability amid changes through a mechanism called allostasis (5). Allostasis maintains stability by detecting both environmental and internal physiological shifts and then triggering tailored adaptive reactions, based on the principle that healthy functioning necessitates ongoing adjustments of the internal physiological environment (5). The stress response involves a complex interaction between the nervous, endocrine, and immune mechanisms, leading to the activation of the sympathetic-adreno-medullary (SAM) axis, the hypothalamic-pituitary-adrenal (HPA) axis, and the immune system (6). Consequently, when one of these systems is activated to maintain allostasis, it often elicits diverse responses across the SAM axis, HPA axis, and the immune system (7). SAM activation triggers the rapid release of norepinephrine and epinephrine, which bind to adrenergic receptors, initiating a cascade of physiological and behavioral responses (8, 9). These include increased heart rate, blood pressure, glucose levels, oxygen consumption, and alertness, while also reducing intestinal motility and promoting vigilance and arousal. The HPA axis response begins with the release of corticotropin-releasing hormone (CRH) from the hypothalamus, which stimulates the anterior pituitary to secrete adrenocorticotropic hormone (ACTH) (10). ACTH then triggers the adrenal cortex to release cortisol, a glucocorticoid that regulates metabolism, immune function, and the stress response (11). The system is modulated by CRH-binding protein (CRH-BP), which helps regulate CRH availability, and pituitary adenylate cyclase-activating polypeptide, which influences CRH production and autonomic stress responses (12).

Prolonged or recurrent exposure to psychosocial stressors is associated with continued activation of the allostatic systems, resulting in adverse physiological effects (13, 14). Allostatic load (AL), a conceptual framework introduced by Bruce McEwen, refers to the cumulative physiological toll of chronic or repeated environmental challenges that are perceived as stressful, leading to sustained or dysregulated neural and neuroendocrine responses (15). AL reflects the cumulative physiological dysregulation that results from ongoing activation of the stress response, often manifesting as sub-clinical alterations across multiple biological systems (4). Such effects are referred to by many as the cumulative “wear and tear” on the body (15). When the demands of the environment surpass one's capacity to manage, it leads to a more severe manifestation, known as allostatic overload (14). This results in a shift towards an intensified state where stress responses are frequently triggered and protective mechanisms fall short (16). Importantly, sources of chronic stress differ markedly across global contexts. For women in low-resource or conflict-affected settings, daily stressors such as food insecurity, environmental degradation, or gender-based violence contribute substantially to physiological wear and tear (17). Integrating these globally relevant stress exposures into allostatic load research can broaden our understanding of how chronic stress shapes maternal health in diverse populations.

Studying the conceptualization of AL rather than isolated physiological systems is crucial for capturing the cumulative and multisystem impact of chronic stress on health outcomes. Conditions that may result in the accumulation or excessive burden of AL include: (1) encountering frequent stressors that could induce chronic stress and recurrent physiological arousal; (2) failure to adapt to recurring stressors; (3) incapacity to deactivate the stress response once the stressor has ceased; (4) insufficient allostatic response to cope with the stressor (13, 18, 19). To understand the effects of such circumstances on the body, we can use AL as a framework to measure physiological responses to acute changes, which result in bodily wear and tear, as well as responses to chronic conditions, which may lead to adverse health outcomes such as diabetes or hypercholesterolemia (17). Varying estimates of AL have been applied in studying humans across a range of developmental and psychosocial outcomes. Research from non-pregnant populations has established associations between chronic perceived stress (20–22), psychological distress (22), depression (18, 23–25), and abnormal illness behavior such as somatization and hypochondriasis (22) with increased AL. A recent systematic review, which synthesized all available evidence up to December 2019 on AL and its influence on health, concluded that higher levels of AL are associated with increased vulnerability to poor health within both clinical and non-clinical adult populations (18). For example, associations were found connecting increased AL levels to poorer self-rated health (26) and increased risk for cardiovascular disease (27). Impaired sleep quality, both in terms of duration and disturbed sleep, was also linked to increased AL (28).

During pregnancy, AL offers a powerful framework for understanding how chronic stress and cumulative physiological burden affect both the birthing person and the developing fetus. Pregnancy is a time of profound and dynamic physiological change, and AL moves beyond the study of single biomarkers to capture the overall “wear and tear” on the body resulting from repeated or prolonged stress exposures. Isolating individual indicators, such as blood pressure or cortisol, fails to account for the complex interactions and synergistic effects across multiple regulatory systems, including cardiovascular, metabolic, immune, and neuroendocrine. In contrast, the AL framework provides a holistic, systems-level perspective that more accurately reflects how stress impacts maternal health and fetal development. Importantly, AL may serve as a biological mechanism underlying adverse perinatal outcomes and may help explain disparities in pregnancy outcomes (29). Emerging research suggests that higher AL, particularly in early pregnancy, is associated with an increased risk of adverse pregnancy outcomes including hypertensive disorders of pregnancy and preterm birth (30). Pregnancy represents a critical developmental inflection point, not only for the birthing person, but also for the fetus. AL provides a conceptual and biological bridge for understanding how maternal stress physiology becomes embedded in the child, aligning closely with the Developmental Origins of Health and Disease (DOHaD) hypothesis (31). Maternal AL may shape fetal brain development, immune function, and metabolic regulation, potentially increasing risk for mental health disorders, cardiovascular disease, and other long-term adverse health outcomes (32). Thus, studying AL during pregnancy allows researchers to move beyond isolated physiological systems to uncover mechanisms of intergenerational transmission of risk. This approach offers valuable insights into the complex, multisystem pathways through which chronic stress affects pregnancy outcomes and can inform the development of targeted strategies to improve maternal and child health across generations.

## 2 Markers and methods for estimating allostatic load in non-pregnant populations

### 2.1 Foundational models and core biomarkers

According to McEwen, AL comprises three types of physiological responses: frequent stress (the magnitude and frequency of response), failed shutdown (chronic activity and the inability to turn off the response), and inadequate response (failure to respond to challenges) (4). In his original paper, McEwen identified the cardiovascular, metabolic, immune, and central nervous systems as the primary systems involved in these physiological responses (15). Seeman et al. (33, 34) identified ten biological parameters in relation to AL: cortisol, dehydroepiandrosterone (DHEA), epinephrine, norepinephrine, total cholesterol (TC), glycosylated hemoglobin (Hb1Ac), resting systolic (SBP) and diastolic blood pressure (DBP), body mass index (BMI), and waist-hip ratio (WHR). The first four parameters (TC, DHEA, epinephrine, and norepinephrine) are considered primary mediators of AL due to their direct correlation with adrenal function, while the remaining parameters (TC, HbA1c, SBP, DBP, BMI, and WHR) are classified as secondary mediators (33, 34). Disease outcomes that result from AL–related pathophysiological processes, such as type 2 diabetes and cardiovascular disease, are referred to as tertiary mediators (13).

Epinephrine and norepinephrine are hormones and neurotransmitters that play key roles in the body's stress response (35). Epinephrine is essential for the acute stress response, known as the “fight or flight” reaction, while norepinephrine is more involved in the “rest and digest” state following the initial surge of adrenaline (35). ACTH and glucocorticoids are critical components of the hypothalamus-pituitary-adrenal (HPA) axis, a major part of the body's stress response system. ACTH stimulates the release of glucocorticoids like cortisol, which help the body manage and adapt to stress, regulate metabolism, and maintain homeostasis (36). Insulin and glucagon work together to maintain glucose homeostasis (37). Insulin lowers blood glucose levels by promoting its uptake and storage, while glucagon raises blood glucose levels by stimulating the release of glucose from storage sites (37). Cytokines, like interleukin-6 (IL-6), are small proteins crucial for cell signaling within the immune system (38). They regulate both innate and adaptive immune responses and can promote or inhibit inflammation (38). Beyond their immunomodulatory roles, cytokines such as IL-6 also influence neuroendocrine function, metabolic regulation, and vascular health, thereby linking immune activation to broader physiological processes (39). Dysregulated cytokine production, particularly sustained elevations in pro-inflammatory cytokines, has been implicated in the pathophysiology of chronic stress, adverse pregnancy outcomes, and long-term disease risk in offspring (40). McEwen also considered health-damaging and health-promoting behaviors as part of the overall concept of allostasis, including smoking, drinking, diet choices, and exercise (13).

### 2.2 Expanding the biomarker battery and methodological challenges

In more recent literature, additional biomarkers such as glucose levels, lipid profiles, and heart rate variability have been recognized for their role in the AL response. These markers have been incorporated into a cumulative index known as the “allostatic load battery” (18, 41) which has proven to be a more accurate predictor of mortality and physical decline than individual biomarkers alone (21, 41–43). Research using this approach has shown that individuals scoring in the highest risk quartiles across multiple biomarkers face increased risk of both cognitive and physiological decline, as well as higher mortality rates (44). However, several limitations arose due to the complexity and dynamic nature of this multisystem network (45).

Given AL's potential as an interdisciplinary index of both personal and public health, a wide range of approaches have been developed to estimate it. Yet the very qualities that make AL unique - its basis in multiple, interacting physiological systems (neuroendocrine, cardiovascular, metabolic, and immune), and its embeddedness within broader socioecological contexts - make it exceptionally challenging to measure. As such, the study of AL is increasingly viewed as a complex systems problem, characterized by nonlinearity, emergence, self-organization, multidimensionality, and network-level interactions (45, 46).

These complex features present several methodological challenges. First, there is no universally accepted biomarker panel or score for operationalizing AL (47). Studies often draw from different sets of indicators, typically based on data availability, which hinders standardization and comparability across research. Second, AL is most quantified as a composite index, where biomarkers are dichotomized according to clinical thresholds or risk quartiles and then summed to produce a total score (42, 48, 49). While this method is straightforward and widely used, it assigns equal weight to all biomarkers regardless of their biological relevance, obscures within- and between-person variability, and limits the ability to examine complex causal pathways between systems. Third, AL may manifest differently across populations. Emerging evidence suggests the existence of distinct AL profiles shaped by differences in socioecological exposures and underlying biological makeup, reinforcing the need for personalized and context-sensitive approaches to measurement (47, 49). Finally, most studies to date have employed cross-sectional designs, restricting insights into how AL evolves over time (45). Longitudinal research is essential to capture the temporal dynamics of AL and its role in shaping health trajectories across the life course.

### 2.3 Quantification of allostatic load

AL is frequently defined using a composite index known as the Allostatic Load Index (ALI), which incorporates biomarkers and anthropometric indicators from multiple physiological systems (50). A common approach for calculating ALI involves the use of empirically derived clinical cut points. One widely used method is based on the National Health and Nutrition Examination Survey (NHANES) (51), in which each biomarker is assigned a score of 0 (within normal range) or 1 (outside the normal range), resulting in a continuous AL summary score. However, inconsistencies in how AL is operationalized, particularly in biomarker selection, cut points, and scoring methods, continue to limit reproducibility and comparability across studies. To address these limitations, novel approaches such as latent profile analysis (LPA) have emerged in AL research. LPA is a novel emerging approach in AL research to elucidate underlying classes of biological dysregulation (23, 52). LPA is a statistical procedure that employs maximum likelihood estimation to determine distinct subgroups based on a set of manifest continuous indicator variables (53–55). This approach allows researchers to detect latent classes of biological dysregulation that may better reflect the heterogeneity and complexity of AL expression across individuals and specific populations.

Importantly, the studies described were conducted in non-pregnant populations, and it remains unclear how these findings translate to pregnancy, where unique physiological changes may alter biomarker patterns and methodological considerations.

## 3 Allostatic load and pregnancy: an overview

### 3.1 Maternal stress and pregnancy outcomes

For nearly twenty years, researchers across health disciplines have attempted to understand the biological mechanisms through which maternal chronic stress affects a range of adverse maternal-infant outcomes. While initial research suggested that stress had minimal impact on pregnancy (56), a substantial body of human studies now indicates that stress, whether mild, moderate, or severe, can negatively affect pregnancy outcomes as well as the behavioral and physiological development of offspring (57, 58). Globally, adverse birth outcomes such as preterm birth and low birth weight remain major public health challenges, accounting for a significant proportion of neonatal deaths, particularly in sub-Saharan Africa and South Asia (59). These outcomes are shaped by intersecting stress exposures, social disadvantage, and structural inequities that differ across settings. Robust literature under the DOHaD framework has demonstrated the role of stress exposure in shaping offspring life course neurobehavioral outcomes through fetal programming mechanisms (60, 61).

Prenatal maternal stress is common among birthing people, with up to 25% reporting high levels of stress during pregnancy (62). This estimate is largely derived from high-income countries, and rates of unreported or unrecognized stress are likely higher in low- and middle-income regions, where stigma, limited screening, and restricted access to mental health services obscure the true prevalence (63). This stress is typically measured by maternal self-report of (1): stressful life experiences, (2) general perceived stress, (3) pregnancy-specific stress, and/or (4) emotional symptoms such as depression or anxiety during pregnancy (64). Research has examined both general and specific types of prenatal maternal stress. For instance, chronic psychosocial stress has been identified as a risk factor for adverse birth outcomes, including preterm birth, low birth weight, and miscarriage (65). Beyond general stressors and living conditions, pregnancy is a unique developmental period with specific stressors related to the pregnancy itself (66, 67). These include concerns about the health of the fetus, worries about physical symptoms, and anxiety about labor and delivery (68, 69). Subjective experiences of these pregnancy-specific stressors are strongly associated with both maternal and fetal outcomes (68, 70). However, most research on prenatal stress and allostatic load has been conducted in the United States and Western Europe, limiting the generalizability of findings. Future studies must include diverse global populations to account for sociocultural variation in stress exposure and coping, as well as differences in healthcare access and maternal morbidity risk.

### 3.2 Allostatic load and adverse pregnancy outcomes

In conjunction with studies that have identified a correlation between chronic stress throughout pregnancy and adverse pregnancy outcomes (71, 72), a burgeoning body of research has explored the relationship between specific AL markers and pregnancy outcomes. Previous studies have highlighted the diverse impacts of AL on various adverse aspects of pregnancy, suggesting the involvement of multiple maternal systems. A study looking at AL biomarkers four months prior to pregnancy found that a unit increase in AL composite scores was associated with increased odds of preeclampsia (62%), preterm birth (44%), and low birth weight (39%) (73). Elevated AL in the first trimester has also been linked to adverse pregnancy outcomes including preterm birth (OR = 1.84, 95% CI: 1.26–12.67) (74), hypertensive disorders of pregnancy (OR = 2.91, 95% CI: 1.50–5.65) (30, 73), and small for gestational age (SGA) for which one study reported a modest, non-significant protective association (OR = 0.8, 95% CI: 0.6–1.0) (29).

Moreover, higher AL, measured by a pregnancy-specific model, has been associated with diminished sleep quality and lower educational attainment, both recognized as chronic stressors (75). High AL during pregnancy has shown correlations with cardiovascular disease risk, hypertension, and metabolic disorders (76). The timing of AL measurement is crucial, as many studies rely on assessments taken before conception or after delivery, which limits their ability to capture changes in AL that occur during pregnancy. Nonetheless, current data suggest that heightened AL during pregnancy plays a significant role in adverse maternal-fetal outcomes.

### 3.3 Physiological changes and implications for AL measurement

Pregnancy is not only an important critical window of development for the fetus, but also for the birthing person. Healthy pregnancies are accompanied by normative linear and non-linear physiological changes across gestation (77). For example, blood pressure decreases until the second trimester, followed by an increase in the third trimester. Total cholesterol and triglycerides rise throughout gestation, while high-density lipoprotein (HDL) increases during the first and second trimesters but decreases in the third trimester (78). A large systematic review found heart rate increases from 79.3 (75.5, 83.1) beats/min at 10 weeks gestation to 86.9 (82.2, 91.6) beats/min at 40 weeks gestation (79) whereas HbA1c levels follow an inverted bell curve across gestation (due to natural insulin resistance) (80). A recent cross-sectional study of AL indicators across gestation in healthy pregnancies documented strong patterns of differences in levels of each individual AL indicator (e.g., SBP and DBP, pulse, BMI, TC, HDL, HbA1c, glucose, and triglycerides) by gestational month (50). When ALI scores were calculated using gestational month–specific risk quartiles, meaning that biomarker risk cutoffs were defined relative to distributions within each gestational month, the resulting ALI scores appeared relatively stable across pregnancy and were not statistically different from those observed in non-pregnant samples (50). Importantly, this does not suggest that physiological functioning remains constant during pregnancy; rather, it highlights that using trimester- or month-specific reference ranges is essential for accurately interpreting AL in the context of the profound and normative biological changes that occur throughout gestation. By demonstrating the feasibility of adapting ALI scoring to account for gestational age, this cross-sectional study supports the utility of AL as a framework for assessing biological dysregulation during pregnancy, provided that gestational timing is appropriately considered.

Pregnancy unfolds as a natural phenomenon characterized by unique physical, physiological, and psychological intricacies within a distinctive social framework. The dynamic interplay of these elements naturally induces an increase in allostatic stress, resulting in the adjustment of various physiological parameters. In addition to the normal basal physiological changes that occur during pregnancy, the physiological stress response systems, which make up AL, are altered compared to the non-pregnant state (81). The purpose of this article is to briefly review results of the latest research on effects of prenatal AL markers across pregnancy. We direct attention specifically to recent research on AL by trimester, to expand on prior review papers summarizing the general knowledge on allostatic load/overload during pregnancy (66, 82) and/or its clinical implications (83, 84). By highlighting these specific sensitive periods of interest, we hope to encourage a synthesis towards a standard metric of ALI during pregnancy and new directions in further research.

## 4 Allostatic load across pregnancy: trimester-specific patterns and outcomes

### 4.1 The first trimester as a critical window: predictive value of early allostatic load for pregnancy and postpartum health outcomes

Several studies focused on the association of various AL markers during the first trimester. Across these studies, AL was computed using some combination of the following biomarkers, based on available data and assays: SBP, DBP, and BMI; serum-measured cholesterol (mg/dl), low-density lipoprotein (LDL; mg/dl), HDL (mg/dl), high-sensitivity C-reactive protein (hsCRP; mg/dl), triglycerides (mg/dl), insulin (μIU/ml), and glucose (mg/dl); and urine-measured creatinine (mg/dl) and albumin (mg/dl) (76).

A study of 4,266 pregnant individuals from the Nulliparous Pregnancy Outcomes Study: Monitoring Mothers-to-Be (nuMoM2b) examined the relationship between AL biomarkers and adverse pregnancy outcomes. Findings revealed that individual components of AL, such as CRP, BMI, DBP, SBP, and HDL, and insulin during the first trimester were significantly associated with hypertensive disorders of pregnancy (OR: 2.5, 95% CI: 2.0–2.9) but not with preterm birth, small for gestational age, or stillbirth (29). High AL during early pregnancy was also associated with 50% greater odds for adverse pregnancy outcomes compared to low AL (29). Using the same cohort, high first-trimester AL was additionally found to be a strong predictor of objectively measured sleep-disordered breathing (SDB), with elevated odds of SDB at any point in pregnancy (aOR = 5.3, 95% CI: 3.6–7.9) and at both early and mid-pregnancy time points (85). These associations were independent of BMI and were attenuated but remained significant when BMI was excluded from the AL score (aOR = 2.6, 95% CI: 1.8–3.9), highlighting a potentially bidirectional link between chronic stress and SDB (85).

Another large-scale longitudinal study assessed the relationship between AL and cardiovascular disease risk 2–7 years postpartum, as well as the pathways contributing to racial disparities in cardiovascular disease risk. High AL in the first trimester was also associated with cardiovascular disease risk, hypertension, and metabolic disorders 2–7 years after delivery (76). In further analyses, the individual components of AL: BMI, DBP, SBP, CRP, and triglycerides, were significantly associated with composite adverse pregnancy outcomes, hypertension, and metabolic disorders, whereas others were not (76). In addition, glucose was associated with metabolic disorder risk (76). Additional work in the same cohort examined whether AL and perceived stress mediated racial and ethnic disparities in hypertensive disorders of pregnancy (HDP). In that analysis, first-trimester AL varied by race and ethnicity and partially mediated the association between being non-Hispanic Black (vs. non-Hispanic White) and HDP (28.9% mediated), whereas perceived stress did not (86). This mediation was not observed for other racial and ethnic groups, highlighting a physiologic pathway through which structural racism may contribute to disparities in HDP (86).

The first trimester may be a critical window for intervention to reduce maternal stress and prevent downstream health complications. The above studies suggest that first-trimester AL may be an early marker for long-term maternal health risks. Certain first-trimester AL biomarkers, notably CRP, BMI, DBP, SBP, HDL, and insulin, are significantly associated with HDP, but not with outcomes like preterm birth, SGA, or stillbirth. However, not all potential stress-related biomarkers (e.g., cortisol, epinephrine, or neuroendocrine markers) were included in these studies. Most studies examining first-trimester allostatic load have been conducted in North American or European populations, limiting the generalizability of findings. Expanding this research to include participants from low- and middle-income countries is crucial to determine whether early-pregnancy physiological responses to chronic stress are consistent across diverse global settings.

### 4.2 The second trimester as a sensitive window: allostatic load as a mid-pregnancy indicator of chronic stress and hypertensive risk

The second trimester is a crucial period where the interplay of physical, hormonal, and psychological factors can significantly influence a pregnant person's AL. Li et al. found the late second trimester may be the optimal time for collecting individual AL indicators, as this period captures the greatest variance in AL levels explained by chronic stress. In this study, the researchers calculated monthly ALI scores across pregnancy using a set of biomarkers spanning cardiovascular, metabolic, inflammatory, and neuroendocrine systems (50). Participants were stratified based on exposure to sociodemographic stressors, and group differences in ALI scores were examined over time (50). The largest differences in ALI scores between those with and without chronic stress exposure were observed at gestational month six, suggesting that mid-pregnancy may be the most informative timepoint for detecting stress-related physiological dysregulation (50). These findings support the idea that the routine six-month prenatal visit may be the optimal time to collect AL indicators when measurement is only feasible once during pregnancy (29, 50).

In the following studies, AL during the second trimester was calculated using some combination of nine components representing three domains of systemic function: SBP, DBP, and pulse pressure for the cardiovascular domain; pre-pregnancy BMI, total cholesterol (TC), HDL, and triglycerides for the metabolic domain; and tumor necrosis factor-alpha (TNF-α) and IL-6 for the inflammatory domain (30, 75).

In a small case-control study, data were drawn from the Prenatal Exposures and Preeclampsia Prevention study, a longitudinal and cross-sectional study conducted between 1997 and 2001. AL was assessed using measurements from plasma samples collected in early pregnancy (before 15 weeks' gestation) from 38 pregnant persons with preeclampsia and 75 matched participants with uncomplicated, term deliveries. The authors found that higher overall AL in the second trimester was associated with increased odds of developing preeclampsia (OR: 2.91, 95% CI: 1.50–5.65), suggesting a possible role of chronic stress in the development of this condition (30). In evaluating the individual domains of AL, only the cardiovascular and metabolic domains were significantly associated with preeclampsia (30). Among the individual components of AL measured before 15 weeks of gestation, systolic blood pressure, diastolic blood pressure, and body mass index were significantly higher among women who later developed preeclampsia (30).

One study analyzed 103 pregnant persons from a low-risk, community-dwelling study population who were enrolled in the Sleep in Pregnancy Study (SLIP) at the University of Pittsburgh. Researchers found that poor sleep quality was associated with increased AL (measured by a pregnancy-specific novel model) in the second trimester. Higher AL scores in early pregnancy were observed among individuals with lower educational attainment, a proxy for socioeconomic status, consistent with patterns seen in non-pregnant populations (75), likely due to reduced access to resources and increased exposure to adverse environmental conditions (e.g., neighborhood deprivation) (13, 42, 87). Overall, depressive symptoms were not found to be correlated with AL in the second trimester (75).

The late second trimester may be the ideal time to assess AL, as it shows the strongest association with chronic stress, likely reflecting the cumulative impact of stress-related physiological changes. During this period, cardiovascular, metabolic, inflammatory, and neuroendocrine adaptations peak, potentially amplifying stress-related dysregulation. However, many findings are based on small case-control studies, which limit generalizability. Nonetheless, second-trimester AL assessment, particularly using cardiovascular and metabolic indicators, could serve as an early biomarker for preeclampsia. Higher AL scores during this time, especially elevated systolic and diastolic blood pressure and body mass index, are significantly associated with increased odds of developing preeclampsia later in pregnancy. Preeclampsia and related hypertensive disorders of pregnancy impact 5%–8% of all births in the United States (88). In the LMICs, severe forms of preeclampsia and eclampsia are more common, ranging from a low of 4% of all deliveries to as high as 18% in parts of Africa and 25% in parts of South America (89). Assessing allostatic load in the late second trimester may allow for early identification of physiological stress, informing strategies to reduce the incidence and severity of preeclampsia. Incorporating such measures into prenatal care could enhance maternal health outcomes globally, particularly in low- and middle-income countries where severe hypertensive disorders are more prevalent.

### 4.3 The third trimester as a period of physiological demand: physiological changes and implications for birth outcomes

AL can be particularly affected during the third trimester of pregnancy as this period involves significant physiological, psychological, and social preparations for the baby's arrival. Throughout these studies, AL was comprised of some combination of eleven components representing multiple systemic functions: SBP, DBP for cardiovascular function; early pregnancy BMI, TC, HbA1c, urinary albumin, and the 1-hour glucose tolerance test for metabolic function; IL-6 and CRP for inflammatory function; and DHEA-S and cortisol for neuroendocrine function (90, 91).

In a community sample, an ALI for 128 pregnant persons was calculated during the third trimester. Upon isolating the components of the ALI, it was found that only cholesterol exhibited a significant and positive correlation with infant length at birth (91). Increased ALI was associated with higher birth weight, birth weight ratio, birth length, and head circumference; however, these associations did not reach statistical significance (91). The only outcome that showed a significant association with ALI was gestational age, with higher ALI levels linked to earlier gestational age in both unadjusted and adjusted models (91).

Of note, a few studies did not observe significant associations between AL calculated during the third trimester and adverse maternal or fetal outcomes. One prospective study, which followed 168 pregnant people from 28 weeks' gestation to delivery, did not find significant associations between AL and birth weight or gestational age (90). In this particular study, there was some variation in the pregnancy-specific components of their measure of AL from those used previously in the literature and included including cardiovascular, metabolic, inflammatory, and kidney function parameters, which may have impacted the findings. Another study including 128 pregnant persons found that gestational age was the only birth outcome of those investigated that appeared to be mildly impacted by AL (92). An additional study conducting secondary analyses of 2,773 participants from the Bogalusa Heart Study (BHS), a biracial (African American/White) study initiated to investigate cardiovascular health in children and young adults, found that AL measured in the third trimester was not associated with preterm birth or low birth weight in fully adjusted models (93).

Findings to date suggest that the third trimester may not be the optimal timepoint for assessing AL in relation to maternal or fetal outcomes. This stage of pregnancy is marked by multiple physiological systems undergoing rapid, simultaneous, and often non-linear changes in preparation for labor. As a result, AL measured during this period may be inherently unstable and difficult to interpret, particularly in studies with small sample sizes where variability can obscure meaningful associations. Earlier stages of pregnancy (e.g., second trimester) may provide a more reliable window for detecting associations with maternal or fetal risk, possibly due to earlier stress-related programming effects on fetal development. Although the third trimester is a period of heightened physiological demand, AL assessed at this time has not consistently predicted birth outcomes such as birth weight, head circumference, or preterm birth. The only somewhat consistent finding is a relationship between higher AL and shorter gestational age; however, even this association is not robust across studies. Among the individual AL biomarkers, cholesterol was the only marker consistently linked to a birth outcome, specifically, infant length at birth. While third-trimester AL may have limited predictive value for immediate birth outcomes, monitoring physiological stress throughout pregnancy can inform strategies to improve maternal and neonatal health. This is especially critical in low- and middle-income countries, where high rates of preterm birth, low birth weight, and limited access to prenatal care make early identification of stress-related risk factors a key component of global maternal health initiatives (63).

### 4.4 Cross-sectional patterns of allostatic load across pregnancy: physiological adaptations and developmental implications

In studies examining AL across pregnancy, from the first to the third trimester, researchers used various combinations of sixteen biomarkers spanning four physiological domains to calculate AL. Cardiovascular markers included SBP, DBP, and pulse pressure; inflammatory markers included CRP, and IL-6; metabolic function was assessed using albumin, TC, HD), creatinine, HbA1c, triglycerides, homocysteine, body BMI, free fatty acids, and serum glucose; and neuroendocrine function was measured using cortisol (32, 50, 94).

One study conducted a secondary analysis using data from 1,056 pregnant people from NHANES, a cross-sectional study designed to assess various aspects of health of adults and children in the United States. Researchers found strong patterns of differences in levels of each individual AL indicator (SBP, DBP, pulse, BMI, TC, HDL, HgbA1c, glucose, and triglycerides) at different gestational months except for CRP (50). Each individual AL indicator changed significantly across gestational months. The pattern of change for each indicator aligned with known pregnancy physiology, suggesting that gestational age should be considered when calculating AL scores in pregnant persons. Pregnant individuals with any sociodemographic stress had higher ALI scores compared to those without sociodemographic stress (50).

In a small exploratory study of 30 mother–child pairs, each interquartile increase in maternal AL during pregnancy was associated with a 25% increase in the child's leukocyte citrate synthase activity and a 15% increase in mtDNA copy number, both indicators of mitochondrial content (32). Additionally, AL was associated with a rise in respiratory chain enzyme activities (16% rise in complex I, a 23% rise in complex II, and a 25% rise in complex IV, which translate to moderate to large effects), indicating an increase in mitochondrial bioenergetic capacity (32). These findings imply that not only is the number of mitochondria affected, but their functional efficiency is also compromised with maternal AL during pregnancy.

Separately, a case-control study using NHANES data found no significant associations between overall AL and birth outcomes such as birth weight or gestational age (94). Interestingly, pregnant individuals exhibited significantly lower mean AL scores (2.3) compared to non-pregnant individuals (2.8; *p* < 0.01), with lower levels of total cholesterol (TC) and higher levels of C-reactive protein (CRP) (94). These findings complement those from other NHANES-based research demonstrating significant, gestational age–related changes in individual AL biomarkers, such as blood pressure, BMI, cholesterol fractions, and glucose metabolism markers, reflecting known physiological adaptations during pregnancy (50). While the case-control study found no direct link between composite AL scores and traditional birth outcomes, the observed shifts in specific biomarkers underscore the complex and dynamic nature of AL during pregnancy.

Moreover, exploratory research into the relationship between maternal AL and offspring mitochondrial function suggests that these physiological changes may have downstream biological consequences beyond birth weight or gestational age (32). Increases in maternal AL have been linked to heightened mitochondrial content and bioenergetic capacity in neonatal leukocytes, pointing to mechanisms by which maternal stress may influence infant cellular metabolism and health. Together, these studies highlight the importance of considering gestational timing and the multidimensional nature of AL when interpreting its impact on perinatal health, suggesting that AL's effects may be mediated through pathways not captured by traditional birth outcomes alone.

There are clear, consistent patterns of change in individual AL markers (e.g., SBP, DBP, glucose, BMI, etc.) across pregnancy that align with known physiological adaptations, emphasizing that gestational age is a critical factor when interpreting AL in pregnancy. Even modest rises in AL are associated with increases in child mitochondrial content and function, suggesting a potential biological embedding of maternal stress through alterations in fetal energy metabolism, highlighting a novel and important developmental pathway. Integrating allostatic load metrics into global maternal surveillance efforts, such as the WHO Maternal and Perinatal Health Database or Demographic and Health Surveys (95), could enable cross-country comparisons and facilitate contextually informed interventions. Emerging evidence linking AL to mitochondrial function in offspring provides a mechanistic insight into how maternal stress might affect long-term child health, this could be a critical pathway in DOHaD.

## 5 Discussion

Across the studies reviewed, the majority consistently report associations between elevated AL and adverse maternal and fetal outcomes. For instance, higher AL in the first and second trimesters is linked to an increased risk of hypertensive disorders of pregnancy and preeclampsia, while elevated third-trimester AL shows a more modest and inconsistent association with outcomes including gestational age and birth weight. This may be because earlier disruptions in maternal physiological regulation have more time to influence placental development and fetal growth trajectories, whereas third-trimester stress may exert more limited effects due to shorter exposure windows or compensatory mechanisms already in place. Moreover, the third trimester is characterized by numerous physiological systems shifting rapidly and in complex, non-linear ways as the body prepares for labor. Assessing AL during this time may therefore reflect a highly variable physiological landscape, which can obscure meaningful patterns, especially in studies with smaller sample sizes, making it harder to identify consistent links with maternal or fetal outcomes.

**Figure 1:**
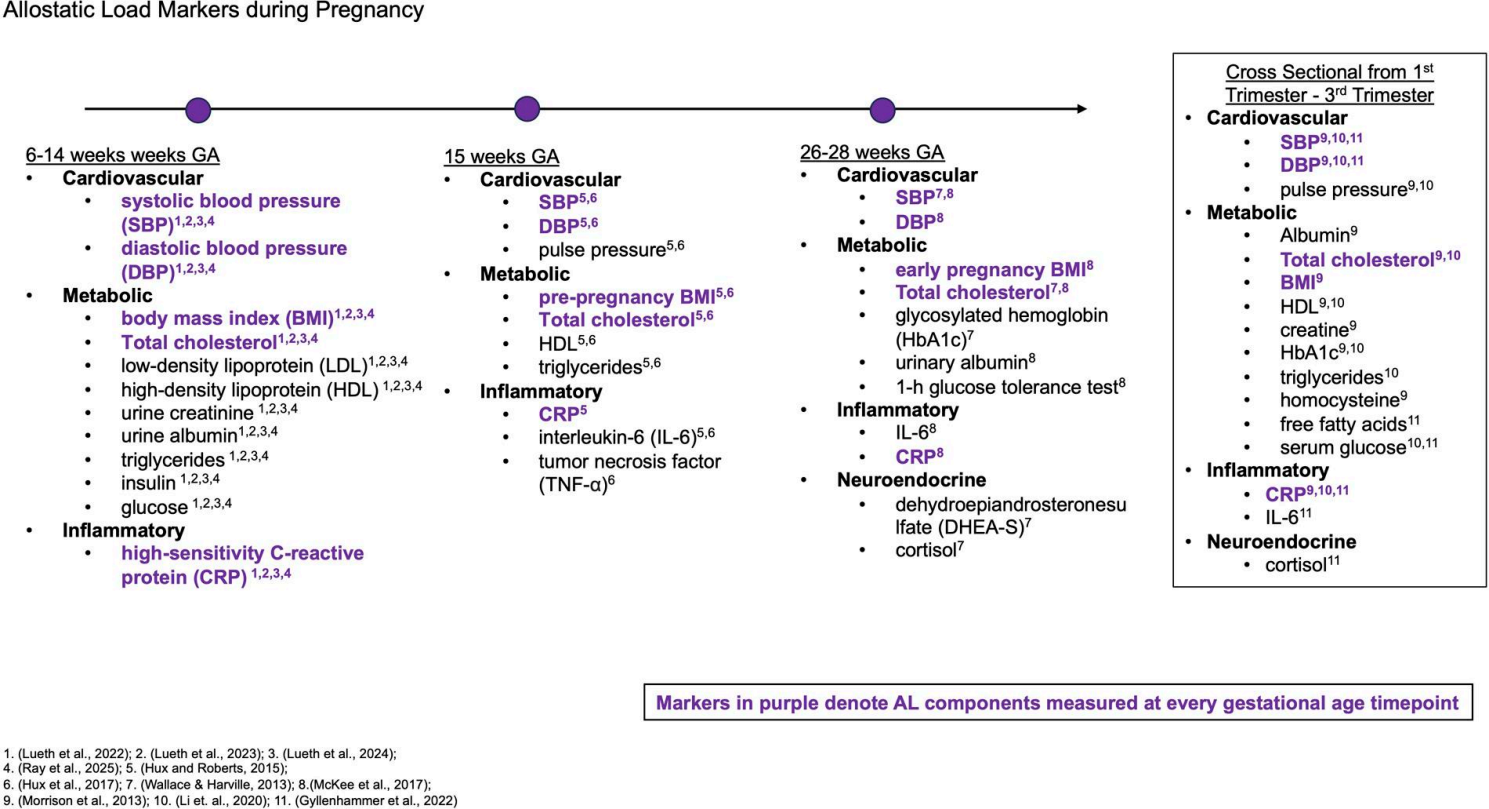
Allostatic load components: figure showing the timing of allostatic load component measurements across gestational age ranges.

Despite emerging patterns, several factors continue to hinder our understanding of AL in pregnancy. For instance, the relevance of assessing AL during pregnancy remains inadequately investigated due to the difficulty in distinguishing between the impacts of chronic stress and the physiological alterations inherent to pregnancy (94). To account for this, some studies have proposed a new stress model that considers the interaction between what a pregnant person perceives as stressful, the coping mechanisms they employ, and the resulting stress-related physiological states (66). There is also considerable inconsistency in how AL is defined and measured during pregnancy (Figure 1). The constructs of AL differ across studies, with variations in the components included and the methods used for estimation. Out of the studies reviewed, only five AL components were consistently studied across gestational age: SBP, DBP, TC, BMI, and CRP. Of the different body systems encompassing AL, only neuroendocrine markers were not consistently measured. Indeed, a review of studies developing a measure for AL revealed inconsistencies in the biomarkers used to define AL (96). Additionally, the criteria for high measures of each biomarker varied. Some studies set cutoff points for high biomarker levels based on clinical standards, whereas others used quartiles specific to their sample as cutoffs.

A key challenge is that there is no consistent “gold standard” methodology for defining AL (21, 97), especially during pregnancy. Central to the AL model is the coordinated function of component biomarkers, which has been demonstrated to offer more consistent predictability compared to individual biomarkers or single-system clusters, especially concerning longer-term outcomes and physical performance (33). Nevertheless, the specific systems included in AL definitions are inconsistent across studies. Among studies reviewed in the present paper, markers related to cardiovascular and metabolic systems were not only prevalent in AL definitions but were also disproportionately represented in ALIs across trimesters compared to other biological systems, mirroring previous review papers (96). Conversely, HPA axis biomarkers (e.g., cortisol, DHEA-S) were assessed during the third trimester and in a cross-sectional gestational age study, only. The absence of HPA axis biomarkers contradicts McEwen and Stellar's original conceptual framework that highlights the significance of these as primary mediators. Indeed, AL is defined as the result of an “heightened neural or neuroendocrine response resulting from repeated or chronic environmental challenge” (15). Despite this, several ALIs now incorporate biological systems not included in the original model. Including multiple systems, especially in the context of pregnancy, is crucial for capturing the complex physiological adaptations occurring across gestation. Cardiovascular, metabolic, inflammatory, and neuroendocrine systems are highly interconnected during pregnancy, and dysregulation in one system can lead to cascading effects in others. A comprehensive, multi-system approach to calculating AL during pregnancy enables a more accurate assessment of cumulative stress-related health risks for both the pregnant individual and the developing fetus. Methods such as latent profile analysis (LPA) or network modeling can be particularly useful for capturing the complex interactions among physiological systems, as they allow researchers to identify distinct patterns of dysregulation and interconnections across cardiovascular, metabolic, and inflammatory markers (98, 99).

Although standardizing the operationalization of AL may not drastically change the observed associations with adverse outcomes, the current lack of methodological coherence makes it difficult to compare findings across studies and time points. This limits our ability to identify which biomarkers during which trimesters are most predictive of risk and ultimately impedes progress in understanding the biological mechanisms through which maternal stress contributes to poor pregnancy outcomes. In addition, there is a lack of long-term intergenerational health consequences and inability to integrate the AL framework into clinical practice. From a global health perspective, research on allostatic load during pregnancy remains heavily concentrated in high-income countries. Few studies have examined AL among women in low- and middle-income contexts, despite these regions carrying the highest burden of adverse pregnancy outcomes. Incorporating global data is essential to capture how socioeconomic instability, climate stressors, and systemic inequities contribute to biological stress accumulation during pregnancy.

There is a pressing need to evaluate the biomarkers, domains, and methodologies used to operationalize AL in pregnant populations, and to determine which constructs are most predictive of adverse perinatal outcomes and long-term maternal health risks. Criticism of measuring AL in pregnancy ascertains that the various biomarkers that comprise AL may reflect proximal factors in pregnancy more strongly than they represent exposure to chronic stress over a pregnant person's lifetime, making conventional AL measures uninformative (91, 94). This raises questions about the informativeness of conventional AL measures during pregnancy. While our review highlights consistent associations between elevated AL and adverse pregnancy outcomes, it also underscores the need for longitudinal studies that track AL both before and throughout pregnancy to disentangle chronic stress effects from pregnancy-specific physiological adaptations. AL may also be inherited, as animal studies have shown that epigenetic changes caused by stress exposure can be passed down through generations (100). However, few human studies have directly examined the long-term consequences of elevated maternal AL on children's later psychopathology, metabolic health, or intergenerational transmission of stress vulnerability, highlighting the need for prospective, longitudinal cohort studies. Future research integrating preconception biomarker data, repeated AL assessments across gestation, and detailed stress exposure histories will be essential to clarify whether AL in pregnancy reflects the cumulative impact of lifelong stress on maternal-infant outcomes or is predominantly driven by normative pregnancy-related physiological changes.

Women with high AL, which reflects the cumulative physiological damage from chronic stress, are at greater risk for complications in future pregnancies, particularly if they experienced such complications previously (101). Similarly, whether elevated AL during pregnancy increases risk for postpartum complications or long-term maternal health outcomes, such as cardiometabolic disease or mental health disorders, remains largely unexplored. In children, maternal elevated AL has been associated with increased risk for neurodevelopmental and psychiatric disorders (102), greater total body fat and abdominal adiposity from birth to early childhood (103), dysregulation of the child's HPA axis (102), and alterations in mitochondrial content and bioenergetic capacity (32). Additionally, studying AL in racially and socioeconomically marginalized pregnant populations, who disproportionately experience chronic stress and health disparities, is essential for understanding and addressing the mechanisms that drive inequities in maternal and child health. One general population study revealed that Black individuals had higher ALI than White individuals at all ages, and lower education and income levels were associated with higher ALI levels across all racial and ethnic groups (42). Additionally, a large recent UK Biobank study of females 40–70 years of age found a 4% increase in average AL for every additional adverse childhood event reported (104).

Developing a standardized, valid method for operationalizing AL in pregnancy studies is critical to ensure that findings are both reliable and reproducible. Future research should explicitly account for gestational age when calculating ALI, as physiological indicators naturally shift throughout pregnancy. Establishing population-based, pregnancy-specific cut-off values for each biomarker at each gestational month will enhance comparability across studies and improve clinical relevance. Evidence suggests that the late second trimester may represent an optimal window for capturing individual AL indicators, given that this period shows the greatest variance in AL levels attributable to chronic stress. To strengthen predictive validity, future studies should adopt multi-system AL assessments at multiple gestational time points, ideally combined with longitudinal follow-up of maternal and infant outcomes. Moreover, research should examine how AL interacts with social determinants of health, psychological stressors, and modifiable lifestyle factors, as understanding these interactions could inform targeted interventions to reduce adverse pregnancy outcomes. Finally, integrating mechanistic biomarkers, such as inflammatory markers or mitochondrial function, may illuminate biological pathways linking maternal stress physiology to perinatal health, providing actionable targets for early prevention and intervention.

### 5.1 Conclusions

Without a strong conceptual basis, it can be difficult to precisely define what AL measures and to interpret findings consistently. While differences in operationalization may not drastically change previously observed associations, the lack of standardization does make it challenging to directly compare results across studies, such as the strength and nature of the relationship between AL and various pregnancy outcomes. Despite this, some important patterns are emerging: elevated AL is consistently linked to adverse maternal and perinatal outcomes, and the specific risks appear to vary depending on the timing of AL assessment during gestation. Broadening this framework to include women across diverse sociocultural and geographic settings will be crucial for advancing global maternal health equity. Developing standardized yet culturally adaptable approaches to assessing allostatic load can bridge gaps between biological and social determinants of health, ultimately improving outcomes for mothers and infants worldwide. These insights underscore the dynamic nature of AL across pregnancy and highlight the potential clinical value of considering gestational timing when measuring AL. Moving forward, developing and adopting a standardized, gestational age–sensitive definition of AL will be critical to improving comparability, enhancing mechanistic understanding, and ultimately, translating this knowledge into effective interventions to support maternal and infant health.
